# Trends in practice intentions and preferences of clinical associate students: Implications for training and health services in South Africa

**DOI:** 10.4102/safp.v62i1.5033

**Published:** 2020-02-06

**Authors:** Saiendhra V. Moodley, Jacqueline E. Wolvaardt, Jakobus M. Louw, Jannie Hugo

**Affiliations:** 1 School of Health Systems and Public Health, Faculty of Health Sciences, University of Pretoria, Pretoria, South Africa; 2 Department of Family Medicine, Faculty of Health Sciences, University of Pretoria, Pretoria, South Africa

**Keywords:** clinical associates, clinical officers, physician assistants, physician associates, practice intentions, practice preferences, study plans, rural practice, migration, district hospitals

## Abstract

**Background:**

The University of Pretoria (UP) had its first intake of Bachelor of Clinical Medical Practice (BCMP) students in 2009. The objectives of this study were to examine the trends in geographical practice intentions and preferences of the first nine cohorts of BCMP students. We also assessed sector and level of care preferences of six BCMP cohorts.

**Methods:**

Cross-sectional studies were conducted 2011, 2014 and 2017. First-, second- and third-year UP BCMP students were invited to complete a electronic questionnaire. Our analyses consisted of calculating proportions for the practice intentions and preferences for each surveys, and performing multiple logistic regression on the aggregated date to determine their associations with sociodemographic and training characteristics.

**Results:**

The proportion of participants intending to practise as a clinical associate in a rural area in South Africa directly after graduating was 62.5% in the 2014 survey and 69.7% in the 2017 survey, compared to 59.6% in the 2011 survey. The majority in all three surveys (53.4% in 2011, 56.6% in 2014 and 59.8% in 2017) indicated a preference for rural practice. Both rural practice intention and rural practice preference were found to be significantly associated with respondent’s self-description of having lived most of her/his life in a rural area, and rural district hospital exposure during training. In 2014 and 2017, approximately two-thirds of the participants selected a public sector option as their most preferred work setting. District hospitals were the most preferred setting of 30.3% participants in 2014 and 32.0% in 2017.

**Conclusion:**

Most participants across the three surveys intended to work in rural settings. Considering that this could provide a sustainable solution to the shortage of health care workforce in rural areas, policy makers in both higher education and health need to promote and ensure the viability of the training of this category of health care providers.

## Introduction

Clinical associates are mid-level medical workers who have completed a 3-year Bachelor of Clinical Medical Practice (BCMP) degree.^1^ This cadre of health workers was introduced in South Africa as one of the country’s strategies to address its human resources for health challenges – particularly the maldistribution between urban and rural areas.^1^ A case study focussing on the Mpumalanga Province suggested that the training and employment of this cadre would be a useful cost-saving strategy to address the demand for health personnel in rural areas without compromising the quality of care.^2^

The University of Pretoria (UP) conducted its first intake of BCMP students in 2009. A survey conducted in 2011 amongst the first three cohorts to enter the BCMP programme at UP found that 59.6% of the participants intended to practise in a rural area following graduation.^3^ As a number of reasons may contribute to intention (e.g. bursary obligations), the students were also asked about their preference.^3^ The preference for working in a rural area was only marginally lower at 53.4%.^3^

The initial survey by Moodley et al.^3^ confirmed that clinical associates could potentially address the shortage of health care workers in rural areas. It was not clear if the BCMP students from subsequent intakes at UP would differ regarding their intentions to practice in rural areas, especially as the results from the initial survey differed markedly from the surveys conducted amongst medical students.^4,5^ These first three intakes could also have differed from those who followed once the degree became known and clinical associates started actual clinical practice. In addition, repeating the surveys could provide some insight on the effect of changes in BCMP selection policy at UP favouring rural-origin students.

The objectives of this study were therefore to examine trends in geographical practice intentions, geographical practice preferences and future study plans of the first nine cohorts of BCMP students at UP by repeating the 2011 survey in 2014 and 2017. An additional objective in 2014 and 2017 was to determine work setting preferences based on sector (public or private or non-governmental organisation [NGO]) and level of care choices. We also aggregated the data from the different surveys in order to determine factors associated with practice intentions and preferences.

## Methods

Three cross-sectional studies were conducted at 3-year intervals in 2011, 2014 and 2017. First-, second- and third-year students registered for the BCMP course at the UP were invited to participate in each of the surveys. All three surveys were conducted in the last quarter (October to December) of the respective years.

Data were collected using a self-administered electronic questionnaire originally developed for the 2011 survey. The questionnaires were delivered using the university’s computer-based training (CBT) platform. Although the CBT platforms in 2014 and 2017 had a different developer (QuestionMark) to the one in 2011 (Umfundi), this did not impact the format of the questionnaire in any significant way. The questionnaire has been described by Moodley et al.^3^ A suitable time when each class would be using the CBT laboratory was identified and the questionnaire was loaded for that session. Students were asked to read through the information about the survey and to give consent electronically. Depending on whether they gave consent or not, the first question would appear on the screen or the programme would terminate.

Our outcome variables included practice intentions directly after graduating from the BCMP programme, the preference to work in South Africa or abroad (if the BCMP degree was internationally recognised), their preference for working in an urban or rural area (if they had complete freedom of choice) and having a public or private sector option (from a list of possible work settings) as their preferred work setting. The sociodemographic variables we considered included gender, age, race, self-description of area they had lived most of their life (urban or rural) and funding source(s) for the degree. The training variables analysed included year of study and rural district hospital exposure during training. In addition, the participants’ interest in pursuing further training was assessed using a series of Likert-scale questions. The ‘strongly agreed’ and ‘agreed’ categories were aggregated for the purposes of our analysis. Our descriptive analyses consisted of calculating proportions for the variables for each of the three surveys.

In order to determine the factors associated with our outcome variables, we aggregated the data from the different surveys. This was done to increase the statistical power. Because of the possibility that some students who had failed would have had an opportunity to complete the survey on two occasions (e.g. a student who failed first year in 2011 would be in third year in 2014), we identified the classes with these students using class registers. In total, 27 students could potentially have completed the survey twice. It was not possible to identify individual students (if any) who completed the survey twice as the survey was anonymous. We, therefore, performed two sets of analyses, namely, one consisting of a reduced data set eliminating the possibility of double counting by excluding certain classes and the other consisting of the full data set. Prior to univariate logistic regression, categories were combined for race (mixed race, Indian, white and other were combined because of their small numbers) and BCMP year (1st and 2nd years were combined). For each of the outcome variables, univariate logistic regression was conducted to determine the association with gender, age, race, self-description of area they had lived most of their life (urban or rural), year of study and rural district hospital exposure during training. In addition, for practice intention, we assessed its association with bursary funding. Independent variables with *p* < 0.25 on univariate logistic regression were included in the multiple logistic regression model for each outcome variable. We then used a backwards stepwise elimination approach for each model using the likelihood-ratio test to obtain the final model for each outcome variable.

We used Stata version 15 (Statacorp; http://www.stata.com) for the 2017 descriptive analyses while the 2014 and 2011 descriptive analyses were conducted using earlier Stata versions. The univariate and multiple logistic regression were conducted using Stata version 15.

### Ethical considerations

The three surveys formed part of an overarching study, ‘A multidisciplinary investigation of authentic learning in the BCMP curriculum’, which was approved by UP’s Faculty of Health Sciences Research Ethics Committee (#56/2011). The survey questionnaire was reviewed and approved by the committee prior to the first survey in 2011. Some modifications to the questionnaire (not affecting the key variables from 2011) were approved by the committee prior to the 2014 survey.

## Results

The proportion of registered BCMP students who participated in the 2014 and 2017 surveys were 48.5% and 58.2%, respectively. Student participation in the three surveys is compared and presented in Table 1. In all three surveys, the proportion of registered third-year BCMP students who participated was greater than 70%. In both the 2014 and 2017 surveys, the proportion of registered first-year BCMP students who participated fell below 40%.

**TABLE 1:** Participation in the 2011, 2014 and 2017 surveys.

Year of study	2011 survey			2014 survey			2017 survey		
	Number of registered students	Students participating		Number of registered students	Students participating		Number of registered students	Students participating	
		*n*	%		*n*	%		*n*	%
BCMP I	77	43	55.8	88	33	37.5	66	24	36.4
BCMP II	92	64	69.6	84	34	40.5	82	51	62.2
BCMP III	47	42	89.4	63	47	74.6	65	49	75.4
All	216	149	69.0	235	114	48.5	213	124	58.2

BCMP, Bachelor of Clinical Medical Practice.

The sociodemographic characteristics of the participants in each of the three surveys are shown in Table 2. In contrast to the 2011 survey, female participants outnumbered male participants in both the 2014 and 2017 surveys. Across all three surveys, the majority of participants were being funded solely by bursaries. Gauteng was identified as their home province (the province where the participant has lived most of her/his life) by the highest proportion of the 2011 survey participants (26.1%). This shifted to Mpumalanga (41.1%) in 2014 and KwaZulu-Natal (48.4%) in 2017. The proportion of participants who had lived most of their lives in a metropolitan municipality was 19.2% in the 2017 survey, which was lower than the previous surveys. A substantially higher proportion of students participating in 2017 considered the area they had lived most of their lives as rural (65.0%) when compared to participants in 2014 (54.0%) and 2011 (50.3%).

**TABLE 2:** Sociodemographic and training characteristics of participants.

Characteristic	2011 Survey		2014 Survey		2017 Survey	
	*n*/*N*	%	*n*/*N*	%	*n*/*N*	%
***Sociodemographic characteristics***						
**Gender**						
Female	73/149	49.0	67/109	61.5	68/123	55.3
Male	76/149	51.0	42/109	38.5	55/123	44.7
**Age**						
18–21 years	59/149	39.6	51/112	45.5	53/124	42.7
≥ 22 years	90/149	60.4	61/112	54.5	71/124	57.3
**Race**						
Black African	131/149	87.9	106/113	93.8	110/123	89.4
Mixed race	4/149	2.7	3/113	2.7	2/123	1.6
Indian	2/149	1.3	1/113	0.9	2/123	1.6
White	11/149	7.4	3/113	2.7	8/123	6.5
Other	1/149	0.7	0/113	0.0	1/123	0.8
**Funding for studies**						
Bursary only	134/149	89.9	96/113	85.0	80/124	64.5
Other source(s)†	15/149	10.1	17/113	15.0	44/124	35.5
**Home province** ‡						
Gauteng	36/138	26.1	14/112	12.5	15/124	12.1
KwaZulu-Natal	28/138	20.3	34/112	30.4	60/124	48.4
Mpumalanga	24/138	17.4	46/112	41.1	22/124	17.7
Limpopo	16/138	11.6	10/112	8.9	16/124	12.9
Other South African provinces	34/138	24.6	7/112	6.3	10/124	8.1
Outside South Africa	0/138	0.0	1/112	0.9	1/124	0.8
**Home municipality** ‡						
District/local	93/136	68.4	86/112	76.8	96/120	80.0
Metropolitan	43/136	31.6	25/112	22.3	23/120	19.2
Outside South Africa	0/136	0.0	1/112	0.9	1/120	0.8
**Rural/urban self-description§**						
Rural	75/149	50.3	61/113	54.0	80/123	65.0
Urban	74/149	49.7	52/113	46.0	43/123	35.0
***Training characteristics***						
**Study year**						
BCMP I	43/149	28.9	33/114	29.0	24/124	19.4
BCMP II	64/149	43.0	34/114	29.8	51/124	41.1
BCMP III	42/149	28.2	47/114	41.2	49/124	39.5
**Rural district hospital exposure during training**						
None	91/148	61.5	57/112	50.9	46/123	37.4
At least some	57/148	38.5	55/112	49.1	77/123	62.6

BCMP, Bachelor of Clinical Medical Practice.

† , Includes those funded by bursaries in combination with other sources.

‡ , Based on where the participant has lived most of her/his life.

§ , Participant perception of whether the area they have lived most of their life is urban or rural.

Similar to the 2011 survey, the majority of participants in both the 2014 (62.5%) and the 2017 (69.7%) surveys intended to practise as a clinical associate in a rural area in South Africa directly after graduating (Table 3). Less than 20% of BCMP students in the 2017 survey intended to practise as a clinical associate in an urban area in South Africa directly after graduating. In each of the surveys, a similar proportion of participants (approximately 10%) intended to remain in South Africa directly after graduating but not practise as a clinical associate.

**TABLE 3:** Geographical practice intentions and practice preferences of Bachelor of Clinical Medical Practice students.

Practice intentions and preferences	2011 Survey		2014 Survey		2017 Survey	
	*n/N*	%	*n/N*	%	*n/N*	%
**Practice intentions directly after graduating**						
Practise as a clinical associate in a rural area in South Africa	87/146	59.6	70/112	62.5	85/122	69.7
Practise as a clinical associate in an urban area in South Africa	41/146	28.1	26/112	23.2	24/122	19.7
Remain in South Africa but not practise as a clinical associate	16/146	11.0	13/112	11.6	12/122	9.8
Emigrate from South Africa	0/146	0.0	2/112	1.8	1/122	0.8
Not intending to complete the BCMP degree	2/146	1.4	1/112	0.9	0/122	0.0
**Practice preference: South Africa or abroad**						
Within South Africa	111/148	75.0	85/113	75.2	89/122	73.0
Outside South Africa	37/148	25.0	28/113	24.8	33/122	27.0
**Practice preference: Urban or rural**						
Rural	79/148	53.4	64/113	56.6	73/122	59.8
Urban	69/148	46.6	49/113	43.4	49/122	40.2

More than 70% of the participants in each of the three surveys indicated a preference for working in South Africa even if their qualification allowed them to work abroad (Table 3). For those preferring to work outside South Africa, the most popular location was the United States in both the 2014 (*n* = 14) and the 2017 (*n* = 13) surveys. Students were asked to indicate an urban or rural practice preference if they had complete freedom of choice. The majority of students in 2014 (56.6%) and 2017 (59.8%) indicated a rural preference as was the case in the survey conducted in 2011.

Approximately two-thirds of the participants in 2014 (67.0%) and 2017 (67.2%) selected a public sector option as their most preferred work setting. District hospitals were selected as the most preferred option by more than 30% of all the participants in 2014 and 2017 (Table 4). A private sector option was selected by 26.6% of the participants in 2014 and 23.8% of the participants in 2017 as their most preferred work setting. Amongst the private sector options, the most popular was owning one’s own private practice. In both 2014 and 2017, less than 10% of participants selected an NGO option as their most preferred work setting.

**TABLE 4:** Sector and level of care practice preferences of Bachelor of Clinical Medical Practice students.

Most preferred setting	2014 Survey *N*=109		2017 Survey *N*=122	
	*n*	%	*n*	%
**Public sector**				
Ward-based outreach team	2	1.8	2	1.6
Primary health care clinic or community health centre	21	19.3	23	18.9
District hospital	33	30.3	39	32.0
Secondary hospital	8	7.3	7	5.7
Tertiary hospital	9	8.3	11	9.0
**Private sector**				
Own private practice	10	9.2	16	13.1
Work with a general practitioner in private practice	5	4.6	3	2.5
Work with a medical specialist in private practice	6	5.5	1	0.8
Private hospital	8	7.3	9	7.4
**NGO sector**				
Work with an NGO in male medical circumcision	4	3.7	4	3.3
Work with an NGO in other medical work	3	2.8	7	5.7

NGO, non-governmental organisation.

We assessed the factors associated with rural practice intention, preference for working in South Africa, rural practice preference and preferred work setting in the public sector using a reduced data set (specified classes excluded to eliminate double counting that may have resulted from students participating in two surveys) and the full data set. Table 5 shows the results for the reduced data set following multiple logistic regression and backwards stepwise elimination. The intention to practice in a rural area as opposed to an urban area was found to be significantly associated with the respondent’s self-description of having lived most of her/his life in a rural area, and rural district hospital exposure during training. Rural practice preference was significantly associated with race, respondent’s self-description of having lived most of her/his life in a rural area, and rural district hospital exposure during training. The preference to practise in South Africa rather than another country was significantly associated with race. The preference to practise in the public sector as opposed to the private sector was found to be significantly associated with age. For rural practice preference, preference to practice in South Africa and public sector preference, the variables found to be significant did not change when we analysed the full data set. In addition to respondent’s self-description of having mostly lived in a rural area and rural district hospital exposure during training, rural practice intention was also found to be significantly associated (odds ratio [OR] = 2.52, *p* = 0.040) with the black African race when we analysed the full data set.

**TABLE 5:** Factors associated with practice intention and preferences.

Characteristic	Odds ratio	95% confidence interval	*p*-value
**Rural practice intention† (*N* = 260)**			
Lived most of their life in a rural area (self-description)	6.23	3.34–11.64	< 0.001
Rural district hospital exposure	3.54	1.89–6.65	< 0.001
**Rural practice preference† (*N* = 301)**			
Black African race	3.71	1.42–9.74	0.008
Lived most of their life in a rural area (self-description)	2.28	1.37–3.82	0.002
Rural district hospital exposure	2.94	1.77–4.89	< 0.001
**South Africa practice preference† (*N* = 303)**			
Black African race	2.85	1.35–6.03	0.006
**Public sector practice preference‡ (*N* = 197)**			
Age ≥ 22 years	2.14	1.16–3.95	0.015

† , Excludes first- and third-year students in 2014.

‡ , No data for 2011 and excludes first-year students in 2014.

The majority of the participants in each of the three surveys indicated that they intended to pursue a further degree or diploma in the future. The proportion of participants intending to pursue further studies (part-time or full-time) was 93.9% (139/148) in the 2011 survey, 84.1% (95/113) in the 2014 survey and 82.6% (100/121) in the 2017 survey. The proportion of participants interested in pursuing a 1-year clinical specialisation was 77.9% in the 2014 survey, which was the highest of the three surveys (Table 6). The 2017 survey saw a lower proportion of participants (45.0%) interested in pursuing a 6-year medical degree following completion of their BCMP degree than the previous two surveys. Interest in a 4-year medical degree was, however, higher amongst participants in 2017 (52.1%) when compared to previous surveys. As in 2011, a high proportion of participants in the two subsequent surveys (41.1% in 2014, 39.8% in 2017) indicated they would consider leaving the BCMP programme prior to completion if they were accepted to study medicine.

**TABLE 6:** Bachelor of Clinical Medical Practice students’ interest in pursuing further training.

Statement	2011 Survey Agree and strongly agree		2014 Survey Agree and strongly agree		2017 Survey Agree and strongly agree	
	*n*/*N*	%	*n*/*N*	%	*n*/*N*	%
I would be interested in pursuing a 1-year specialisation in a specific clinical discipline (e.g. orthopaedics, obstetrics) following completion of my BCMP degree	94/146	64.4	88/113	77.9	85/122	69.7
I would be interested in pursuing a diploma in public health or community-oriented primary care	-	N/A†	70/112	62.5	66/121	54.5
I am interested in pursuing a 6-year medical degree (MBChB/MBBS) following completion of my BCMP degree	74/147	50.3	62/113	54.9	54/120	45.0
I am interested in pursuing a 4-year medical degree (e.g. 4-year MBBS programme at Wits) following completion of my BCMP degree	68/146	46.6	54/112	48.2	63/121	52.1
I would consider leaving the BCMP programme prior to completion if I was accepted to study medicine (MBChB/MBBS)	62/146	42.5	46/112	41.1	47/118	39.8

BCMP, Bachelor of Clinical Medical Practice; Wits, University of the Witwatersrand; MBChB/MBBS, Bachelor of Medicine, Bachelor of Surgery.

† , This question was not part of the 2011 questionnaire. Included in 2014 for the first time.

## Discussion

The proportion of BCMP students intending to practice as clinical associates in a rural area in South Africa was found to be substantial in our 2011 survey at approximately 60%.^3^ This proportion is higher than noted in a study conducted amongst physician assistants in the United States where the authors reported that 39% of participants predicted rural practice.^6^ Our 2014 and 2017 surveys found even higher rural practice intention proportions, with almost 70% in 2017 indicating an intention to practice in a rural area directly after graduating. A follow-up study of the first four UP cohorts showed that the practice intentions of BCMP students do translate into actual practice choices, as 53% of them were practising as clinical associates in rural settings.^7^ The link between rural practice intentions and rural practice has also been confirmed by Shannon and Jackson.^6^ who reported that over 70% of the respondents who intended to work in a rural site, did so. In order for rural practice intention to translate into actual rural practice, an adequate number of clinical associate posts need to be created and funded at rural health facilities by provincial governments in South Africa. It is not clear whether this has been done and presents a lost opportunity to address rural workforce shortages.

The proportion of students who intend to practice in a rural area is a result (at least in part) of the large number of BCMP students from rural areas. A number of studies amongst medical students have found a link between rural practice intention and rural background.^8,9,10,11^ The link between rural practice intention and rural background was also reported amongst physician assistants in the United States.^6,12^ In our multivariable analysis, we found that rural practice intention was significantly associated with self-description of having lived most of one’s life in a rural area. The increase in rural practice intention seen in 2017 may be explained by the fact that the proportion of participants self-describing their background as rural was 65.0% in 2017, compared to 50.3% in 2011.

The increase in rural-origin students was achieved by purposeful selection. As from the 2014 first-year cohort, the calculation of the merit point score used in the selection of BCMP students at UP included additional points for rural students on the basis of their rural origin and rural schooling. Furthermore, since 2010, seats in the programme are allocated according to the provinces from which applicants originate, and provinces with large rural populations are allocated more seats. Both these measures reduce the chances of urban students displacing rural students from the selection list. Our results suggest that changes in the UP BCMP selection policy have achieved one of its desired outcomes of providing a sustainable rural health workforce.

A plausible explanation for the large number of BCMP students intending to practice in rural areas directly after qualifying is that the majority were solely funded by bursaries. Bursaries are usually linked to mandatory employment post-qualification in the funding province (where provincial governments provided the bursary). However, being funded solely by a bursary was not found to be significantly associated with rural practice intentions in our multivariable analysis. We repeated our analysis combining those with full and partial bursaries and, again, did not find a significant association. Bursaries, therefore, did not explain the high proportions of students intending to practise in rural areas. Based on our overall findings, provincial governments (and other bursary funders) wanting to address rural workforce shortages should be directing their bursaries to students from rural backgrounds.

Practice preferences (the choice they would make independent of obligations such as bursaries, family, etc.) indicated a majority preferring rural practice in all three surveys, suggesting a substantial proportion of the BCMP students had an intrinsic motivation to work in rural areas. While findings by Burch et al.^4^ (in a study in which more than half the participants were South African) suggest that rural practice is favoured by a small minority of South African medical students, a more recent study conducted at three South African universities by Naidu et al.^13^ reported that 52% of medical students and 38% of non-medical health sciences students prefer rural practice.

In our study, gender was not associated with intention to practice in a rural setting. Similarly, there was no association between current practice choice and gender in the study by Monareng et al.^7^ The lack of association between gender and practice choice, or by extension intention to practice, differs from what has been previously reported.^12^ Unlike gender, the respondents’ race was significantly associated with both a preference to practise in South Africa and a preference to practise in a rural area. Ethnicity was found to play a role in rural practice choice in only a single study reviewed by Wilson et al.,^14^ and it is not clear why black African students in our study were more likely to have this preference. This finding probably does not warrant any change in recruitment policy as black African students already make up more than 85% of the BCMP student population at UP.

In addition to a rural background, we found that both rural practice intention and preference were significantly associated with rural district hospital exposure during training. This is consistent with the literature, as there is some evidence that the inclusion of a rural rotation in the curriculum of medical students has a positive impact on influencing rural practice choice.^14^ Currently, at least half of the second- and third-year clinical associate students at UP are placed in rural district hospitals. Apart from an initial 2 weeks and tests and examinations in Pretoria, they spend the whole year in their respective clinical placements. Our evidence suggests that rural district hospital exposure should be incorporated in the training time of as many BCMP students as possible both at UP and at other institutions.

Migration is not considered to pose a significant threat to South Africa’s clinical associate workforce, as the qualification is not currently automatically accepted internationally for registration to practice. In the event it were to be internationally accepted, a clear majority of participants (above 70%) in each of the three surveys indicated a preference for remaining and working in South Africa. This finding is in contrast to a study on the migration intentions of South African medical and nursing students.^15^ George et al.^15^ found 58.9% of medical students and 66.6% of nursing students at three South African medical schools and a single nursing school, respectively, intended to migrate within 5 years of qualification. Intention of clinical associates to emigrate could change once in the workplace as the follow-up study of the first four UP cohorts reported that the intention to emigrate rose to 31% once in the workplace.^7^

We found that the majority of UP BCMP students (more than 60%) would opt for a public sector facility as their most preferred work setting. In comparison, only 28% of South African medical students in the study conducted by Burch et al.^4^ intended to practise in the public sector in the long term. De Vries et al.^8^ found that significantly more final-year medical students planned to practise for the majority of their working lives in the private sector as opposed to the public sector. Naidu et al.^13^ reported that private hospitals were the most popular institution choice amongst non-medical health sciences students in their study while district hospitals were the most popular choice amongst medical students. District hospitals were the preference of more than 30% of all participants in our study in 2014 and 2017. This is not surprising as this is where the majority of their practical training takes place. The most popular private sector option amongst our participants in 2014 and 2017 was their own private practice. In terms of their scope of practice, clinical associates are not permitted to have their own private practice.^16^

Across all three surveys, more than 80% of participants planned on further studies. A one year post-qualification clinical specialisation was a popular choice in all three surveys. Currently, only the University of the Witwatersrand has met this training need with a honours degree in emergency medicine for clinical associates. Specialisations in anaesthesia and obstetrics are currently being explored by various role-players. All three surveys found a high proportion of participants interested in pursuing a medical degree. While the 6-year medical degree was the more popular option in 2011 and 2014, the 4-year medical degree was the more popular option in 2017. The reason for this change is unclear but may be a function of the information they have about the entry requirements and selection process for the 4-year degree.

The limitations with respect to the first survey have been discussed by Moodley et al.^3^ In all three surveys, students were not provided with a definition of ‘urban’ or ‘rural’, and the responses therefore represent their understanding of the terms. The problems with the definitions of these terms have also been reported by Shannon and Jackson^6^ who used population size, which also proved problematic. While participation from the first- and second-year students in the 2014 and 2017 surveys was not as high as we would have liked, there is no reason to believe that those who did not participate differed systematically from the participants with respect to practice intentions and preferences. There was the possibility that a small number of students participated in two surveys, which meant that aggregating the data for the different surveys for purposes of determining factors associated with intentions and preferences may have led to double counting. To address this, we excluded specific classes for this part of the analysis to eliminate that possibility. We also performed the logistic regression using the full data set to confirm if our main findings would remain the same. While generalisation of our findings to other BCMP programmes in South Africa and equivalent programmes in Africa should be done with caution given the differences in selection policies and training, we feel there is sufficient evidence for recruitment policies directed at BCMP students from rural backgrounds and for rural placements during their training.

## Conclusion

The three surveys have confirmed that a majority of BCMP students at UP intend to practise in rural areas immediately following graduation and have a longer-term rural practice preference. Both rural practice intention and rural practice preference proportions increased over the three surveys, reflecting the increasing proportion of rural students in the programme. The use of purposeful selection criteria clearly results in achieving the goal of recruiting rural-origin students, and by extension the provision of a rural workforce.

Participants had strong aspirations to study further and the interest to study medicine has not reduced over the nine cohorts of students. The interest to study medicine remains a concern, and if universities do not respond to the need for clinical specialisation options – especially those most needed by rural populations – these students are likely to explore other options. Clinical associates can provide a sustainable solution to South Africa’s rural health workforce shortages if adequate numbers of funded posts are created and if their career aspirations can be met. These students are clearly willing to meet the health needs of rural populations, but it will require political will to ensure that they will be able to do so.
